# *Culex quinquefasciatus* Mosquitoes Resist Acquisition of Methicillin-Resistant *Staphylococcus aureus*: Insights from Field and Laboratory Studies

**DOI:** 10.3390/antibiotics13070618

**Published:** 2024-07-02

**Authors:** Waseema Arif, Gowdham Thangaraj, Pushpa Srinivasan, Srikanth Srirama, Panneer Devaraju

**Affiliations:** 1 Unit of One Health, ICMR-Vector Control Research Centre, Puducherry 605006, India; 2 Public Health Entomology, ICMR-Vector Control Research Centre, Puducherry 605006, India

**Keywords:** antimicrobial resistance, environment, insects, mosquitoes, *Culex quinquefasciatus*, methicillin-resistant *Staphylococcus aureus*

## Abstract

The emergence of antimicrobial resistance (AMR) in pathogens and their spillover into the environment have become a global public health menace. Insects can acquire these pathogens from the environment and would serve as mechanical and biological vectors. The current study assessed the ability of *Culex quinquefasciatus* mosquitoes to acquire methicillin-resistant *Staphylococcus aureus* (MRSA) through the exposure of the mosquitoes to the pathogen via rearing water, blood feed, or a feeding membrane under laboratory conditions. In addition, mosquito immatures collected from their habitat in the vicinity of hospitals, veterinary dispensaries, and butcher shops at 15 study sites in Puducherry were screened for MRSA infection. All samples were subjected to the culture-based isolation of *S. aureus* from the surface and homogenate. The presence of the *S. aureus*-specific *nuc* gene and the genes that confer resistance to methicillin (*mecA* and *mecC*) were screened using PCR tests. MRSA was not evident either on the external surface or in the homogenate of the mosquitoes emerging from the larvae reared in the MRSA-spiked water or those fed with MRSA through blood or smeared membranes. Furthermore, the presence of MRSA was not evident in any of the field-caught mosquitoes. Hence, we conclude that *C. quinquefasciatus* mosquitoes are impervious to MRSA colonization.

## 1. Introduction

Antimicrobial resistance (AMR) is recognized as a major threat to public health and is estimated to cause 10 million deaths annually by 2050 [1]. It refers to the ability of microorganisms to resist antimicrobials through the acquisition of resistant genes conferring AMR. The spillover of AMR pathogens into the environment is evident by reports of their presence in environmental compartments including soil, wastewater systems, groundwater, aerosols, wild animals, and surfaces in contact with humans [2]. In addition, reports on the evolution of new AMR pathogens by the horizontal transfer of the genes are on the rise [3]. Infections with such AMR pathogens render mainstay antibiotics ineffective, prolong the mean period of hospitalization, and increase mortality rates [4]. Consequently, the World Health Organization (WHO) has convened the Global Leaders Group on antimicrobial resistance and emphasized AMR as a One Health problem (WHO/EMP/IAU/2018.05). India tops the list of countries with high antibiotic consumption levels and has been actively involved in AMR research and surveillance [5]. The Indian Council of Medical Research (ICMR) has reported a steady decline in the antibiotic susceptibility of locally isolated ESKAPE pathogens (*Enterococcus faecium*, *Staphylococcus aureus*, *Klebsiella pneumoniae*, *Acinetobacter baumannii*, *Pseudomonas aeruginosa*, and *Enterobacter* spp.) in recent years [6]. The lack of stewardship of antibiotic use in agriculture, livestock production, and human and animal healthcare is considered the major factor contributing to the development and dissemination of AMR into the environment. It affects human, animal, and environmental health. Hence, a “One Health” approach involving surveillance, research on the mechanisms of spread and acquisition of resistance, and integrated stewardship on the use and disposal of antibiotics in human and animal health care systems is in the need to combat the transmission and spillover of AMR genes [7].

*Staphylococci* are a group of Gram-positive cocci that reside in the skin and mucous membranes of mammals. They are recognized as a major opportunistic pathogen of humans causing skin and soft tissue infections, endocarditis, osteomyelitis, pneumonia, meningitis, gastroenteritis, and urinary tract and prosthetic device infections [8]. The emergence of methicillin-resistant *Staphylococci* (MRS), majorly *S. aureus* (MRSA), has led to longer hospitalizations, severe complications, high morbidity, and mortality [9]. The observed resistance has been attributed to the acquisition of *mecA* and *mecC* through the horizontal transfer of the mobile genetic element *Staphylococcal cassette chromosome mec* (*SCCmec*) [10]. MRSA accounts for up to 50% of *S. aureus* bloodstream infections in certain parts of Asia. Notably, Japan and South Korea exhibit a high prevalence, with MRSA accounting for over 70% of clinical isolates [11]. In India, the rise in the prevalence of MRSA has been reported from 28.4% in 2016 to 42.6% in 2021 across different regional centers [6]. In Puducherry, a union territory in the southern region of India, 30 of 450 (6.67%) human clinical samples analyzed in a study were identified to have MRSA [12].

Although MRSA is considered a nosocomial pathogen, community-acquired infections are steadily increasing. Reports on the prevalence of MRSA in various nonclinical environments have been on the rise in the recent past, signifying them as a major public health threat [13]. A yearlong MRSA surveillance project at an equine hospital reported 8.6% of surface samples including computers, water buckets, and surgery tables, to be positive for MRSA, suggesting them as hotspots [14]. A high genetic relatedness was identified between 17 MRSA strains isolated from the environment surrounding 15 MRSA patients, signifying the environment as a reservoir for MRSA [15]. Further, MRSA has also been reported in municipal wastewaters [16] and synanthropic animals (rodents) [17], indicating the widespread spill of the pathogen into the environment.

Insects are well-established vectors of viral, bacterial, and several other parasitic infections. In recent studies, certain insects were identified as reservoirs for AMR bacteria, favoring horizontal gene transfer, and few were identified as unlikely vectors for AMR. In addition, the vertical transmission of AMR genes from one generation to another and mechanical transfer through legs, mouthparts, and feces have also been reported [3]. Interestingly, the surface of *Anopheles* mosquito was reported to include pathogens of human infection, indicating the risk of mechanical transfer to humans [18]. In the case of MRSA, insects including houseflies, cockroaches, and bedbugs collected from hospitals have been reported as possible mechanical and biological vectors for MRSA [19,20,21]. However, there are no reports on the dissemination of MRSA by mosquitoes. Hence, in this study, we have carried out (i) experiments to expose immature and adult *Culex quinquefasciatus* mosquitoes to MRSA to determine their ability to acquire the pathogen and (ii) the screening of MRSA in the external surface and homogenate of mosquito immatures collected from environmental wastewaters to validate the experimental results.

## 2. Results

### 2.1. Phenotypic and Molecular Characterization of MRSA

**Figure 1:**
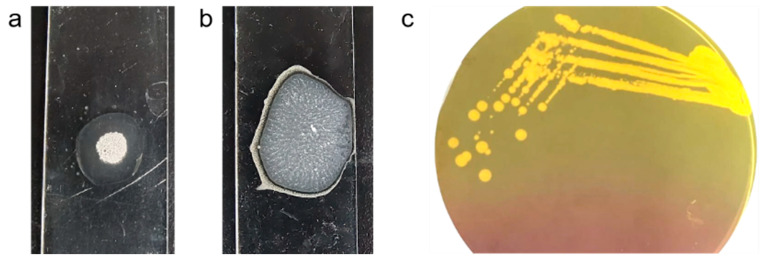
*S. aureus* cultured in mannitol salt agar media producing golden yellow colonies (**a**) and exhibiting positivity in catalase (**b**) and coagulase (**c**) test.

**Figure 2:**
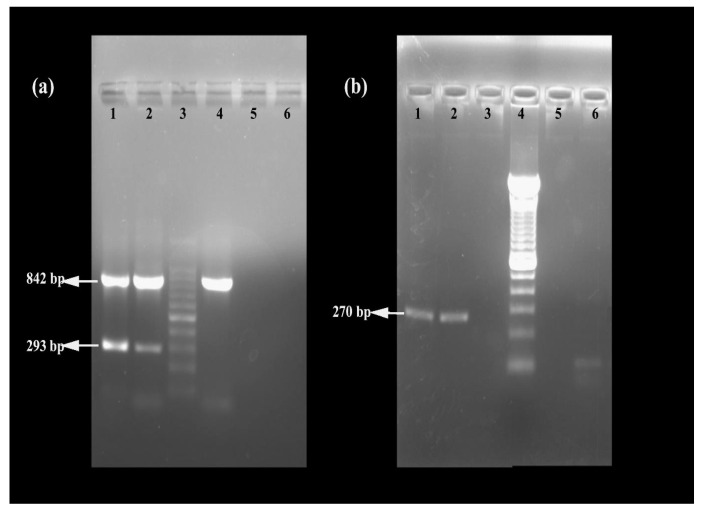
Confirmation of MRSA using (**a**) multiplex PCR targeting *16SrRNA*, *mecA*, and *mecC* genes (lane 1: positive control, lane 2: MRSA strain used in the study, lane 3: 100 bp DNA ladder, lane 4: methicillin-sensitive *S. aureus*, lane 5: negative control, and lane 6: unused well) and (**b**) PCR targeting *nuc* gene (lane 1: positive control, lane 2: MRSA strain used in the study, lane 3: negative control, lane 4: 100 bp DNA ladder, and lane 5 and 6: bacterial cultures which turned out to be negative for *nuc*).

**Figure 3:**
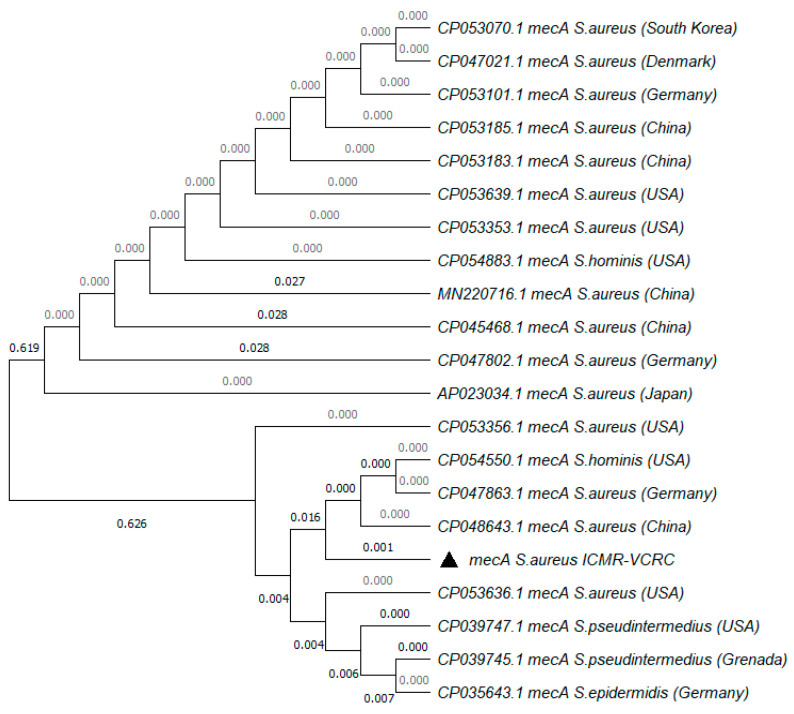
Phylogenetic tree of *mecA* gene amplified from MRSA strain used in the study. The tree was constructed with 1000 bootstrap replicates using the maximum likelihood method. The triangle symbol indicates the strain used in the current study.

**Figure 4:**
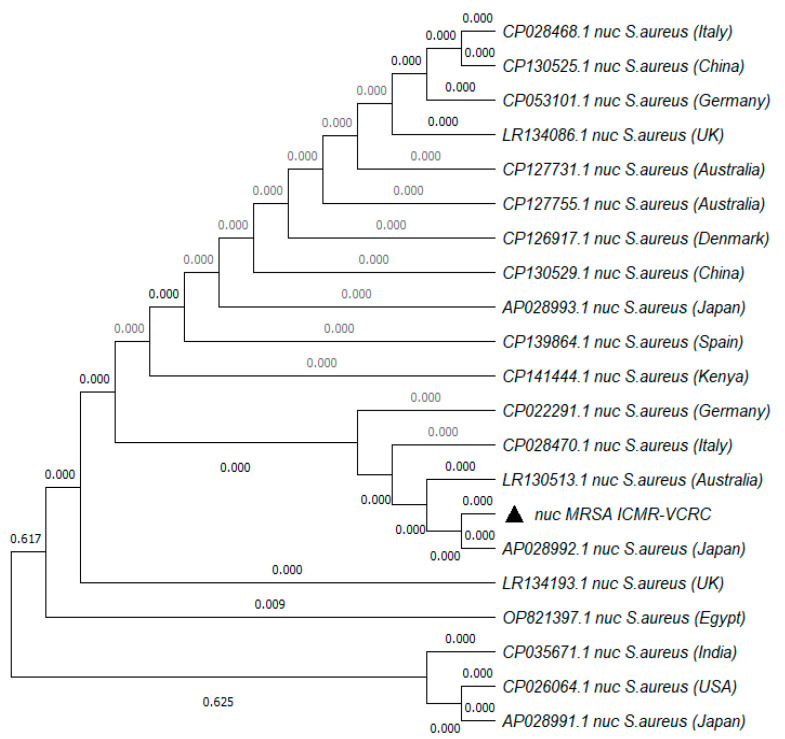
Phylogenetic tree of *nuc* gene amplified from MRSA strain used in the study. The tree was constructed with 1000 bootstrap replicates using the maximum likelihood method. The triangle symbol indicates the strain used in the current study.

The MRSA strain utilized for the experiments was characterized as Gram-positive cocci that tested positive using both catalase and coagulase tests (Figure 1a,b). The bacterial strain exhibited mannitol fermentation, forming yellow colonies on mannitol salt agar media (Figure 1c). Further, the strain demonstrated positivity in the cefoxitin screen and displayed resistance to oxacillin (≥4 MIC) in the antibiotic susceptibility test and was consequently confirmed as MRSA. The antibiogram of the strain indicated its resistance to other antimicrobials such as benzylpenicillin, ciprofloxacin, levofloxacin, erythromycin, clindamycin, and trimethoprim (Table 1). Resistance to methicillin was further confirmed with a disk diffusion assay using a cefoxitin-impregnated disk [22]. Using dilution plating, the bacterial concentration was identified to be 6.1 × 10^7^ CFU/mL. The PCR amplification of the *mecA* gene associated with resistance to methicillin, and the *nuc* gene specific to *S. aureus* was evident in the DNA extracted from the culture (Figure 2a,b). The nucleotide sequencing of the resulting amplicons confirmed the presence of *mecA* and *nuc* genes. Phylogenetic trees constructed based on the sequences from *mecA* and *nuc* genes are depicted in Figure 3 and Figure 4, respectively.

### 2.2. The Inability of Mosquitoes to Acquire MRSA

Laboratory-reared *C. quinquefasciatus* larvae, pupae, and adult mosquitoes used for the study were tested to be free of MRSA on the external surface and in the homogenate. When these larvae were reared in MRSA-spiked water, the external surface wash of 33.33% of larval pools collected on the 1st and 3rd day post-exposure (dpe) tested positive for MRSA using multiplex PCR assay. However, MRSA was not detected in the external surface wash samples of larvae, pupae, or adults collected on the 5th, 7th, and 14th dpe, or in the homogenate samples collected on all sampling days (Table 2). Subsequent adult feeding experiments using MRSA-spiked blood or a feeding membrane smeared with the bacteria resulted in MRSA positivity in the homogenate of 33.33% of mosquito pools collected only on the 1st day post-feeding (dpf). MRSA was not detected in the homogenate samples of adults collected on the 3rd, 5th, 7th, and 14th dpf, or on the external surface wash samples collected on all sampling days (Table 2).

### 2.3. Absence of MRSA in Field-Caught Mosquito Immatures

**Figure 5:**
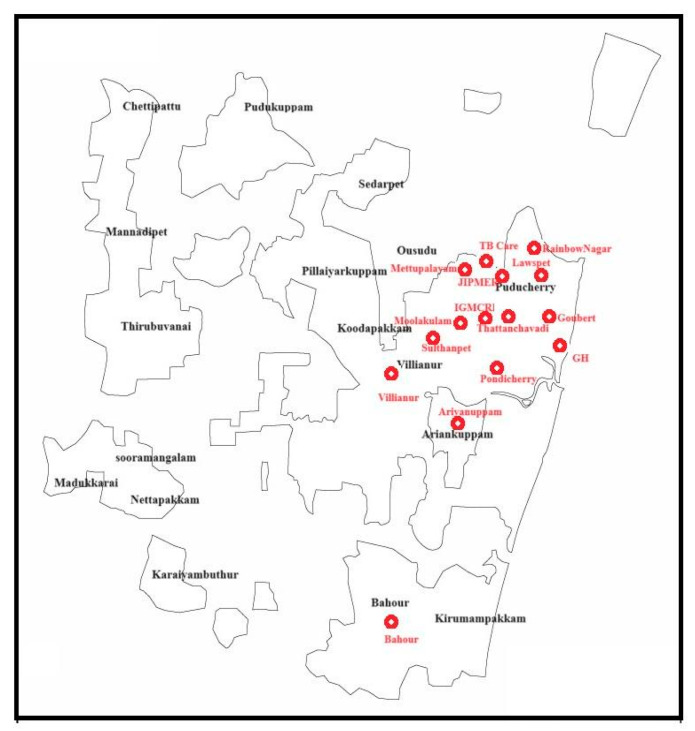
Geographical map of Puducherry depicting the study sites.

A total of 802 (518 larvae and 284 pupae) mosquito immatures were collected from wastewater drains in the vicinity of hospitals, veterinary dispensaries, and butcher shops at 15 study sites in Puducherry (Figure 5). They were confirmed to be *C. quinquefasciatus* based on the morpho-taxonomical analysis. Among them, external surface wash and homogenate samples of 48 larval pools and 48 pupal pools were screened for MRSA. The amplification of the *16S rRNA* gene specific to *Staphylococcus* sp. was observed in 54.2% of the larval surface, 12.5% of the larval homogenate, 39.58% of the pupal surface, and 2.08% of the pupal homogenate samples (Table 3). However, the PCR amplification of *nuc*, *mecA*, and *mecC* genes was not evident in any of the samples screened.

## 3. Discussion

Globally, the prevalence of MRSA is increasing in hospital settings by the nosocomial route and in community settings by the spillover of the pathogen into the environment from humans and animals [13]. In addition to surfaces, wastewater has been identified as a significant reservoir of MRSA. Studies conducted in the USA revealed that 50% of the wastewater samples collected from four treatment plants harbored MRSA [23]. Similarly, a research study conducted in Sweden identified genetic similarities between MRSA isolated from wastewater and clinical samples, suggesting the possible spillover from the community [16]. In India, *S. aureus* strains with complete resistance to methicillin were isolated from municipal wastewater in the state of Punjab [24]. In addition to the environmental reservoirs, insects are considered sentinels of AMR that drive the transmission of resistant pathogens to newer geographic territories [3]. Over the past decade, studies have identified MRSA in bedbugs and cockroaches recovered from hospitals and reported them as potential mechanical and biological vectors for the dissemination of MRSA [20,21]. Furthermore, MRSA detected in the external surface of stable flies (7%) and house flies (27%) collected from a pig farm, persisted for 96 h after removal from the farm, signifying them as potent mechanical vectors [19]. Mosquitoes, being holometabolous insects with three aquatic life stages, acquire microflora from their natural habitats [25]. The immatures of mosquitoes breeding in MRSA-contaminated wastewater drains may acquire the pathogen from their habitat. In addition, during a bloodmeal, mosquitoes can acquire microflora from the host’s skin and bloodstream [18]. Hence, this study was carried out to experimentally expose the larvae and adults of mosquitoes to MRSA and assess their susceptibility to MRSA colonization. All the experiments involved *C. quinquefasciatus* mosquitoes as they breed in sewages and wastewaters. Such habitats support a diverse microbiome including MRSA, thereby increasing the risk of pathogen acquisition by the mosquito immatures breeding in them [16,23,24]. In addition, the mosquito immatures breeding in wastewater drains located in the 15 selected study sites were screened for MRSA to validate the experimental findings. These field-caught mosquito immatures were purposively sampled from wastewater drains in the vicinity of hospitals and veterinary dispensaries, to ensure their exposure to spillover MRSA from the above sources.

The MRSA strain utilized in the study met criteria for confirmation as MRSA according to CSLI standards [26]. The *nuc* and *mecA* genes amplified from the culture were significantly similar to the known sequences in the NCBI database (Figure 3 and Figure 4). The presence of *mecA* and the absence of *mecC* in the culture indicated the role of low-affinity penicillin-binding protein (PBP2A) in conferring resistance to β-lactam antibiotics [27]. According to previous reports, the concentration of MRSA typically ranges from 10^2^ to 10^3^ CFU/mL in sewage and 10^5^ to 10^6^ CFU/mL in the skin of humans infected with MRSA [28,29]. Hence, to simulate the larvae rearing in a natural habitat contaminated with MRSA, the rearing water was spiked with 1 × 10^3^ CFU/mL of MRSA. In order to ensure the availability of viable bacteria, the rearing water was replaced on alternate days and spiked with fresh culture of MRSA. Similarly, to determine the ability of mosquitoes to acquire MRSA from the bloodstream or skin of the feeding host, blood spiked with 10^6^ CFU/mL or a membrane smeared with 10^6^ CFU/mL of the bacteria were used for feeding. At the same concentration, MRSA was observed to colonize bedbugs [30].The spiking was performed just before feeding to ensure the viability of the bacteria.

In the current study, MRSA was detected on the external surface of only the larval samples and not on pupae or adults, highlighting a weaker role for mosquitoes as mechanical vectors of MRSA. This may be attributed to the adherence of the bacteria to the exoskeleton facilitated by the setae present predominantly on the surface of the larvae. These hair-like structures primarily aid in locomotion and sensory perception [31]. However, upon molting, larvae shed off their outer exoskeleton to become pupae. Pupae then undergo metamorphosis, and the adult mosquitoes emerge by splitting the pupal skin. Unlike larval exoskeletons, pupal and adult surfaces have fewer setae as the need for locomotion and sensory perception is reduced in the pupae and is supported by wings and antennae in adults [32]. This reduction in hair-like structures on the exoskeleton may be associated with the observed ability of pupae and adults to resist the colonization of MRSA on the surface.

In the tissue homogenates analyzed, MRSA positivity was restricted to the homogenate of the 1st dpf samples of adults fed with MRSA via spiked blood or bacterial smeared membranes. MRSA was not observed in the homogenate of adult mosquitoes sampled on subsequent days post-feeding and in the homogenate of any of the immatures experimentally exposed to MRSA. Similarly, none of the mosquito immatures collected from wastewater drains in Puducherry tested positive for MRSA. These observations indicate that the internal tissues of mosquitoes, irrespective of the life stage, are impervious to MRSA, highlighting them as unlikely biological vectors of MRSA. These findings are in contrast to the previous reports of MRSA colonization in insects such as bedbugs, cockroaches, and houseflies, where both the external surface and internal tissues were colonized by the organism [19,20,21]. Similarly, the presence of MRSA was not observed in the wild *C. quinquefasciatus* mosquitoes collected from the study sites in Puducherry. Although there are no published reports on the prevalence of MRSA in the environmental samples of the study area, we have identified the presence of MRSA with *mecA* and *mecC* genetic elements in rodents and shrews trapped from these sites in our previous study [33]. This observation highlighted the spillover of MRSA in the environment. The absence of MRSA in the immatures collected from wastewater in the vicinity of hospitals, veterinary dispensaries, and butcher shops in these sites reflects the imperviousness of *C. quinquefasciatus* to MRSA. Recently, Herrara et al. (2023), demonstrated that bedbugs can acquire MRSA from an infected experimental feeding membrane, maintain it, and transmit it to a sterile membrane during subsequent feeding [30]. In the case of *C. quinquefasciatus* mosquitoes, such phenomena were not observed in our study. However, our findings are similar to the report on common house spiders by Baxtrom et al. (2006), where MRSA was not identified either in the external or internal microbial flora of more than 100 screened spiders [34]. This difference in the susceptibility of insects to MRSA may be attributed to multiple factors including the insect’s feeding behavior, immune modulations, and diverse endogenous microflora that could restrict the growth of MRSA [35]. Mosquitoes can elicit strong cellular and humoral immune responses that might have led to lysis or the hemocyte-mediated phagocytosis of MRSA [36,37]. The gut microbes of the mosquitoes engaged in symbiotic relationships might have established colonization resistance by physical competition [38]. In addition, the genetic burden behind the acquisition of resistance genes can also alter several characteristics of the bacteria [39]. But the above mechanisms are to be explored for confirmation. Recently, Abu-Hussien et al. (2024) have isolated 65 *S. aureus* strains from *C. pipiens* collected from three study sites in the Cairo governorate of Egypt, of which 18 strains were resistant to tetracycline, 20 to gentamicin, and 12 to ciprofloxacin [40]. However, our feeding experiments only included MRSA. In our study, *S. aureus* isolates sensitive to methicillin or resistant to other antimicrobials were not analyzed. This may be considered a limitation of the study. Interestingly, although the presence of *S. aureus* in *C. quinquefasciatus*, as reported by Abu-Hussien et al. (2024), was not evident in our mosquito population, *Staphylococcus non-aureus* (*16SrRNA* positive and *nuc* negative) was observed in our laboratory-reared and field-caught (54.2% of larval surface, 12.5% of larval homogenate, 39.58% of pupal surface, and 2.08% of pupal homogenate) *C. quinquefasciatus* mosquitoes. Hence, we acknowledge the need for further studies to understand the role played by such endogenous *Staphylococcus* species present in the mosquitoes in rendering the mosquito resistant to MRSA colonization.

## 4. Conclusions

To our knowledge, this is the first study to analyze the ability of MRSA to colonize *C. quinquefasciatus* mosquitoes. We conclude that (i) the absence of MRSA in the external surface and lack of persistence in the internal tissues of *C. quinquefasciatus* mosquitoes experimentally exposed to MRSA through a breeding habitat and feeding, and (ii) the absence of MRSA in the mosquito immatures collected from their natural habitats in Puducherry, signify the resistance of these mosquitoes to MRSA colonization. The study was designed only to identify the ability of mosquitoes to acquire MRSA and follow-up studies should concentrate on deciphering the actual mechanism behind the observed imperviousness to MRSA.

## 5. Materials and Methods

### 5.1. C. quinquefasciatus Colony

Adults and immatures of *C. quinquefasciatus* mosquitoes were obtained from the rearing and colonization facility at the Indian Council of Medical Research—Vector Control Research Centre (ICMR-VCRC). The larvae were maintained in ceramic larval trays and fed with larval food (crushed Tetramin tablets, Tetra, Blacksburg, VA, USA). Emerged adults were maintained in one-foot Barraud cages (30 cm × 30 cm × 30 cm) at 28 ± 2 °C temperature and a relative humidity of 70–80%. Adult mosquitoes were fed with a 10% sugar solution soaked in cotton wool. Female mosquitoes were fed chicken blood once per week using an artificial membrane feeding system and were allowed to oviposit. The study was approved by the Institutional Biosafety Committee of the institute (ICMRVCRC/IBSC/2303).

### 5.2. MRSA Culture

MRSA culture was maintained in sterile Luria broth containing 7.5% sodium chloride in an incubator set at 37 °C. Species confirmation was carried out by Gram’s staining, and catalase and coagulase tests. For the catalase test, a loop full of bacteria was smeared onto a clean glass slide and incubated with a drop of 3% hydrogen peroxide. The observation of effervescence/bubbles due to the release of oxygen indicated the presence of catalase enzymes in the bacteria (Figure 1a). For the coagulase test, a loop full of bacteria was smeared onto a clean glass slide and incubated with a few drops of human plasma. The formation of fibrin clots indicated the presence of coagulase enzymes in the colony (Figure 1b). Further, a loop of bacteria was streaked onto differential media containing mannitol salt agar and was incubated overnight at 37 °C. The formation of golden yellow colonies confirmed mannitol fermentation (Figure 1c).

### 5.3. Phenotypic Characterization of MRSA

Antibiotic resistance was profiled using automated VITEK-2 AST-P628 antibiotic susceptibility testing cards. A single bacterial colony was diluted in a sterile saline solution and was processed as per the manufacturer’s instruction. VITEK-2 COMPACT (Biomerieux, Durham, NC, USA) was used for the assay. Resistance to methicillin was further confirmed with a disk diffusion assay using disks impregnated with cefoxitin (30 μg) [22].

### 5.4. Molecular Characterization of MRSA

The culture was subjected to the molecular detection of the *S. aureus*-specific *nuc* (thermonuclease) gene and genetic elements conferring methicillin resistance, viz., *mecA* and *mecC*. DNA from the culture established from a single colony of *S. aureus* was extracted by boiling and the snap chill method [41]. Briefly, the bacterial pellet was washed thrice in 0.01 M phosphate buffer saline (PBS), and the pellet was reconstituted in 500 µL of PBS with 1% Triton X-100. The bacterial suspension was boiled for 10 min, followed by snap chilling for 10 min. The bacterial suspension was then centrifuged, and the supernatant was used as the DNA template. PCR targeting the *nuc* gene was carried out to confirm the growth of *S. aureus* [42]. Multiplex PCR targeting *mecA* and *mecC* genes was performed as described by Venugopal et al. (2020) to screen for the presence of genetic elements conferring methicillin resistance along with *16S rRNA* as an internal control for PCR [43]. The primer sequences and PCR cycling conditions are described in Table 4. The amplicons were subjected to Sanger sequencing in an Applied Biosystems Genetic Analyzer 3130XL and the phylogenetic tree was constructed with 1000 bootstrap replicates using the maximum likelihood method in MEGA v.7.0 software [44].

### 5.5. Dilution Plating

Colony-forming units of MRSA per mL (CFU/mL) of the media were determined with dilution plating onto Mannitol salt agar plates [45]. Briefly, the bacterial culture was subjected to 10-fold serial dilution in sterile PBS and 100 µL of sample from each dilution was pour-plated onto sterile Mannitol salt agar plates. The plates were incubated at 37 °C for 24 h. The plates with countable number of colonies were enumerated and CFU/mL was determined [45] using the formula given below:

CFU/mL = (number of colonies × dilution factor)/volume of culture plated in mL.

### 5.6. Experimental Exposure of MRSA to Larvae

Six larval trays, each with seventy L1 larvae, were included in the experiment. The larvae were reared either in water spiked with MRSA (10^3^ CFU/mL) or normal water without MRSA in triplicates (*n* = 3 tray/group). In both groups, rearing water with and without MRSA, respectively, was replaced on alternate days. Larval feed was provided on a routine basis and daily mortality was monitored. Five larvae per tray were sampled on the 1st, the 3rd, and 5th dpe, 5 pupae per tray were sampled on the 7th dpe, and five adults emerging from the pupae were collected on the 14th dpe. All the samples were screened for MRSA acquisition using selective enrichment and multiplex PCR.

### 5.7. Experimental Feeding of MRSA to Adults

Red blood cells from whole chicken blood were isolated using centrifugation, the RBC concentrate was washed thrice with sterile PBS, and 5 mM of ATP was used as a phagostimulant. RBC concentrate and bacterial culture mixed at a 1:3 ratio was used for feeding. Nine cages, each with 70 adult females, were left to starve overnight. Mosquitoes were fed either with (i) MRSA-spiked blood (10^6^ CFU/mL) through a sterile membrane (Hemotek, Blackburn, UK), (ii) normal blood through a membrane smeared with 10^6^ CFU/mL of MRSA as described by Herrara et al. (2023) [30], or (iii) normal blood through a sterile membrane (*n* = 3 cages/group). The blood-fed mosquitoes were maintained in 10% sucrose and monitored for mortality daily. Five adults per cage were sampled on the 1st, 3rd, 5th, 7th, and 14th dpf and screened for the acquisition of MRSA.

### 5.8. Field Collection of Mosquito Immatures

Purposive sampling was carried out in fifteen sites in Puducherry Union Territory. In each site, wastewater drains in the vicinity of hospitals, veterinary dispensaries, and butcher shops were examined for the presence of mosquito immatures. The mosquito immatures were collected using sterile dippers and transported to the laboratory in sterile containers. Larvae were subjected to taxonomical identification using standard keys [31] and segregated into pools (five immatures constituted a pool).

### 5.9. Screening for MRSA

Each pool of mosquito immatures/adults was rinsed in 500 μL of sterile 0.01 M PBS four times. All four wash solutions of the respective sample were pooled together to analyze the presence of MRSA on the external surface. The surface sterilization of the larva/adult was achieved by rinsing it in 70% ethanol for 30 s and the residual ethanol was removed by washing in PBS thrice. Further, the samples were homogenized in 500 μL of sterile PBS using tissue lyser (Qiagen, Hilden, Germany). *S. aureus* in the external surface wash, and homogenates of mosquito immatures and adults, were subjected to selective enrichment. Briefly, 100 µL of each sample was inoculated in sterile Luria broth containing 7.5% sodium chloride for primary enrichment and incubated at 37 °C for 24 h. The enriched cultures were streaked on Mannitol salt agar (MSA) plates and incubated overnight at 37 °C. Yellow colonies indicating mannitol fermentation confirmed the presence of *S. aureus*. PCR was used for targeting the *nuc* gene and multiplex PCR was used for the molecular confirmation of *S. aureus* and genes conferring methicillin resistance, respectively.

## Figures and Tables

**Table 1:** Antibiotic susceptibility of the MRSA strain used in the study.

Antimicrobial	MIC (µg/mL)	Interpretation	Antimicrobial	MIC (µg/mL)	Interpretation
Cefoxitin Screen	≥4	R	Linezolid	2	S
Benzylpenicillin	≥0.5	R	Daptomycin	0.5	S
Oxacillin	≥4	R	Teicoplanin	≤0.5	S
Gentamicin	4	S	Tetracycline	≤1	S
Ciprofloxacin	≥8	R	Tigecycline	≤0.12	S
Levofloxacin	4	R	Nitrofurantoin	≤16	S
Inducible Clindamycin Resistance	+	+	Rifampicin	≤0.03	S
Erythromycin	≥8	R	Trimethoprim	80	R
Clindamycin	0.25	R	Vancomycin	≤0.5	S

MIC: minimum Inhibitory concentration, R: resistant, S: susceptible and +: positive.

**Table 2:** Screening of MRSA in the external surface wash and homogenate samples of mosquito immatures and adults fed with MRSA.

Feeding	Day 1		Day 3		Day 5		Day 7		Day 14	
	Ex	Hm	Ex	Hm	Ex	Hm	Ex	Hm	Ex	Hm
Larvae reared in MRSA-spiked water	+ (1/3)	−	+ (1/3)	−	−	−	−	−	−	−
Larvae reared in unspiked water	−	−	−	−	−	−	−	−	−	−
Adults fed with MRSA-spiked blood through a sterile membrane	−	+ (1/3)	−	−	−	−	−	−	−	−
Adults fed with unspiked blood through MRSA-smeared membrane	−	+ (1/3)	−	−	−	−	−	−	−	−
Adults fed with unspiked blood through a sterile membrane	−	−	−	−	−	−	−	−	−	−

The number within the bracket indicates the fraction of larval pools positive for MRSA using PCR. “Ex” and “Hm” represent the external surface wash and homogenate of the mosquitoes, respectively. “+” indicates positivity for MRSA.

**Table 3:** Screening of MRSA in field-caught mosquito immatures.

S. No.	Sample	Larvae		Pupae	
		External Surface (*n* = 48 Pools)	Homogenate (*n* = 48 Pools)	External Surface (*n* = 48 Pools)	Homogenate (*n* = 48 Pools)
1	*16SrRNA* ^+^	26	6	19	1
2	*mec A* ^+^	0	0	0	0
3	*nuc* ^+^	0	0	0	0

“+” indicates positivity for the gene.

**Table 4:** Details of primers and PCR cycling conditions used for molecular characterization of MRSA.

S. No	Genes	Oligonucleotide Sequence (5′–3′)	Cycling Conditions
1	*16S rRNA* (842 bp)	FP: GTGATCGGCCACACTGGA RP:CAACTTAATGATGGCAACTAAGC	Initial denaturation: 94 °C, 5 min Denaturation: 94 °C, 1 min Annealing: 52.5 °C, 1 min Elongation: 72 °C, 1 min Final elongation: 72 °C, 10 min
2	*mecA* (293 bp)	FP: ACGAGTAGATGCTCAATATAA RP: CTTAGTTCTTTAGCGATTGC	
3	*mecC* (584 bp)	FP: GCTCCTAATGCTAATGCA RP: GGCTTAGAACGCCTCTATGA	
4	*nuc* (270 bp)	FP: GCGATTGATGGTGATACGGT RP:AGCCAAGCCTTGACGAACTAAAGC	Initial denaturation: 95 °C, 10 min Denaturation: 94 °C, 1 min Annealing: 55 °C, 30 s Elongation: 72 °C, 1.5 min Final elongation: 72 °C, 5 min

## Data Availability

The original contributions presented in the study are included in the article and further inquiries can be directed to the corresponding author.
